# Adapting a health video library for use in Afghanistan: provider-level acceptability and lessons for strengthening operational feasibility

**DOI:** 10.1186/s12960-020-00477-9

**Published:** 2020-05-19

**Authors:** Lara Lorenzetti, Jenae Tharaldson, Subarna Pradhan, Sayed Haroon Rastagar, Shafiqullah Hemat, Sharif A. H. Ahmadzai, Lisa S. Dulli, Amy Weissman, Catherine S. Todd

**Affiliations:** 1 Global Health, Population and Nutrition, FHI 360, Durham, NC USA; 2 FHI 360, HEMAYAT Project, Kabul, Afghanistan; 3 Health Promotions Department, Ministry of Public Health, Islamic Republic of Afghanistan, Kabul, Afghanistan; 4 Asia Pacific Regional Office, FHI 360, Bangkok, Thailand

**Keywords:** Afghanistan, Community health workers, Maternal health, Demand generation, Social and behavior change, Counseling

## Abstract

**Background:**

Community health workers (CHWs) in Afghanistan are a critical care extender for primary health services, including reproductive, maternal, neonatal, and child health (RMNCH) care. However, volunteer CHWs face challenges including an ever-expanding number of tasks and insufficient time to conduct them. We piloted a health video library (HVL) intervention, a tablet-based tool to improve health promotion and counseling by CHWs. We qualitatively assessed provider-level acceptability and operational feasibility.

**Methods:**

CHWs implemented the HVL pilot in three rural districts of Balkh, Herat, and Kandahar provinces. We employed qualitative methods, conducting 47 in-depth interviews (IDIs) with male and female CHWs and six IDIs with community health supervisors. We used semi-structured interview guides to explore provider perceptions of program implementation processes and solicit feedback on how to improve the HVL intervention to inform scale-up. We conducted a thematic analysis.

**Results:**

CHWs reported that the HVL increased time efficiencies, reduced work burden, and enhanced professional credibility within their communities. CHWs felt video content and format were accessible for low literacy clients, but also identified challenges to operational feasibility. Although tablets were considered easy-to-use, certain technical issues required continued support from supervisors and family. Charging tablets was difficult due to inconsistent electricity access. Although some CHWs reported reaching most households in their catchment area for visits with the HVL, others were unable to visit all households due to sizeable populations and gender-related barriers, including women’s limited mobility.

**Conclusions:**

The HVL was acceptable and feasible for integration into existing CHW duties, indicating it may improve RMNCH counseling, contributing to increased care-seeking behaviors in Afghanistan. Short-term challenges with technology and hardware can be addressed through continued training and provision of solar chargers. Longer-term challenges, including tablet costs, community coverage, and gender issues, require further consideration with an emphasis on equitable distribution.

## Introduction

With a predominantly rural population, Afghanistan struggles with a low health workforce density [1, 2]. Geographic imbalances in formal health worker distribution, particularly for female providers, are pronounced in areas facing insecurity or socio-cultural barriers limiting coverage [2]. This presents a major challenge for delivering health services in Afghanistan, which has the highest maternal and infant mortality ratios in Asia, despite a decline over the past 15 years [3]. Consequently, community health workers (CHWs) are fundamental to health service provision in Afghanistan, especially for women and children [4]. These volunteers provide health promotion, basic care, and referrals to more than 70% of the population [5]. In Afghanistan, there are an estimated 19,000–28,000 CHWs [6], of which approximately one third are women. Most CHWs are married and have low levels of literacy [2, 7]. After being appointed through community health *shura* (leadership committee) nominations, CHWs complete a 4-month pre-service training course, then receive 3-day refresher trainings every 6 months [2]. Community health supervisors (CHSs) are predominantly male, public-sector employees assigned to supervise and train CHWs working at health posts within the catchment area of their assigned health facility.

Globally, CHWs have contributed to a variety of reproductive, maternal, neonatal, and child health (RMNCH) outcomes, including reduced maternal, infant, and child morbidity and mortality [8–10]; improved modern contraceptive uptake [8, 11]; increased immunization coverage [8, 12]; and promotion of exclusive breastfeeding [8, 13]. Female CHWs are critical for RMNCH care in Afghanistan, as the presence of female CHWs has been associated with increased antenatal care, skilled birth attendance, and modern contraceptive use [14]. Although more than half of Afghan CHWs are women [4], their ability to serve as CHWs depends on customs allowing women to work and move freely [5]. Moreover, female and male CHWs often face issues of time poverty [15] and lack of professional credibility in some settings [4]. Demanding workloads may reduce retention, as CHWs are expected to assume an ever-increasing number of tasks despite their unpaid status [4]. An informal CHW replacement program has worked to fill service gaps, though many replacements do not receive necessary basic training [4].

To address some of these challenges, mobile health technologies have been used to facilitate CHW-provided services [16]. Pilot studies of tablets and mobile phone counseling interventions found that mobile technologies improved CHW engagement with clients [17, 18]. Videos also allowed CHWs to multi-task, making more efficient use of their time [19]. To enhance CHW-led community-based RMNCH counseling and service demand creation efforts, we developed and pilot-tested the tablet-based health video library (HVL) intervention, which was implemented by CHWs in three rural districts of Afghanistan. The overall goal of this cross-sectional, mixed-methods assessment was to describe the feasibility and acceptability of the HVL for providers and their clients. Here we report exclusively on qualitative findings from in-depth interviews (IDIs) with providers (i.e., CHWs and their respective CHSs). The aim of this qualitative assessment was to examine whether the HVL was operationally feasible and perceived as acceptable for daily work by CHWs, who directly implemented the HVL. We used provider interviews to assess their perceptions of short- and long-term operational challenges to HVL implementation and generate suggestions for program improvements to facilitate HVL scale-up.

## Methods

A larger cross-sectional, mixed methods study, which included this qualitative analysis, was approved by Afghanistan’s Ministry of Public Health (MOPH) Institutional Review Board (#44010) and exempted by FHI 360’s Office of International Research Ethics. The equal mixed methods approach (quant+qual) consisted of IDIs with providers, which were conducted concurrent with a structured household survey of randomly selected married men and women in communities where the HVL was implemented. The qualitative component was intended to assess the operational feasibility and provider-level acceptability of the HVL, complementing the assessment of client-level feasibility and acceptability data collected through the structured survey.

### Health video library pilot setting and providers

The USAID-funded Helping Mothers and Children Thrive in Afghanistan (HEMAYAT) project collaborated with the MOPH to develop and test the HVL intervention. The HVL was designed as a tool for CHWs to use to facilitate counseling and generate client uptake of healthy behaviors and demand for facility-based services. Beginning in July 2017, the HVL pilot was implemented over approximately 6 months in three geographically and culturally distinct rural districts: Char-Kent in Balkh Province, Karukh in Herat Province, and Daman in Kandahar Province. Char-Kent District is in the north-central part of the country, where Dari and Uzbek are the predominant languages spoken. Karukh District is in the western portion of Afghanistan with both Dari and Pashto spoken and with close trading relationships to Iran that influence norms. In Daman District in southern Afghanistan, the predominant spoken language is Pashto.

CHWs and their affiliated health posts were purposively selected for pilot participation and subsequent assessment based on their status as a health post with a currently active female CHW in the catchment area of a HEMAYAT-supported health facility. We required current CHW activity (confirmed by CHS monitoring records) due to high CHW attrition rates and stipulated having an active female CHW given that only female CHWs could engage the initial HVL target audience—pregnant women and mothers of children under 5 years—based on social norms [2]. Men have not been widely included in RMNCH interventions in Afghanistan, despite being the primary health decision-makers within families [20]; thus, we wanted to have a general comparison of the effect of including male CHWs on the ability to reach men. We initially excluded male CHWs from Herat to make these comparisons.

### HVL training and implementation support

For the pilot activity, we trained and provided selected CHWs with tablets loaded with a “video library,” a collection of brief pre-tested videos in Dari and Pashto delivering dramatizations of key RMNCH behaviors and services (Table 1) followed by CHW-led counseling and referrals. We trained ten female CHWs per district, each from a different health post, totaling 30 female CHWs and their respective health posts. In Balkh and Kandahar, 10 male CHWs from the same health posts were also trained and instructed to share the tablet with their female counterpart, who was assigned primary custody. By male CHW demand, we informally trained 10 male CHWs from the same health posts in Herat approximately 2 months into the pilot. We also trained the CHS assigned to the health facility from whose catchment area the CHW group was selected. HEMAYAT staff conducted HVL training over 1 day, comprising both didactic (e.g., how to use a tablet, main counseling points following each video) and practical components.

**Table 1:** Video topics included in pilot phase

• Benefits of early breastfeeding/colostrum	
• Exclusive breastfeeding/harms of bottle feeding	
• Child vaccination schedules	
• Chlorhexidine for umbilical cord care	
• Contraceptive implant use	
• Harms associated with early marriage	
• Lactational amenorrhea method	
• Maternal tetanus vaccination	
• Obstetric fistula prevention and treatment	
• Postpartum hemorrhage prevention	
• Postpartum intrauterine device use	
• Respectful maternity care	
• Women’s right to health care	
• Other (non-specific)	

Trained CHWs then returned to their communities and engaged clients with HVL presentations and counseling during group sessions at health posts or home visits. They targeted audiences of married women, who were pregnant or had at least one child under 5 years of age, and household key influencers (i.e., husbands and mothers-in-law). Staff from the HEMAYAT project and the CHS conducted a field mentorship visit within the first 3 weeks of HVL implementation, then monthly visits to observe HVL and counseling sessions. They provided technical assistance and informal refresher training as needed, focusing on tablet use and review of video content, counseling techniques and points, and indicated actions (e.g., referrals). CHWs were also given telephone numbers of Kabul-based HEMAYAT staff trainers and were encouraged to call with any technical issues, particularly those related to tablet operation. No formal refresher training was provided during the pilot period.

### Sampling, recruitment, and data collection for qualitative assessment

Between February and April 2018, we invited for interview all CHWs and CHSs involved in the HVL pilot in each district, including male CHWs in Herat informally trained during monitoring visits. Ten CHWs of each sex per district were invited for interview, consistent with guidance that 6 to 12 IDIs per homogenous group is sufficient for reaching ≥ 80% thematic saturation [21]. CHWs were invited to a refresher training where the assessment was presented by sex-matched staff trained in qualitative interview techniques and human subjects research. CHWs provided verbal informed consent, and audio-recorded interviews were conducted over approximately 45 min in Dari or Pashto in a private room at the training venue.

Interviews were facilitated using a 15-question semi-structured guide that explored feasibility and acceptability of the HVL for providers. Feasibility was conceptualized as CHWs’ ability to use the HVL and the extent that it improved their workflow and confidence. Acceptability was defined as provider perceptions of whether the HVL was beneficial to their ability to engage and counsel clients and their professional standing within the community. Questions focused on five main areas that map to feasibility, acceptability, or both: (1) experience delivering the intervention, (2) intervention features that providers liked or disliked, (3) barriers and facilitators to HVL use, (4) motivation for intervention delivery, and (5) strategies to improve the intervention and recommended future topics. Audio-recordings were transcribed in Dari or Pashto, then translated into English for analysis.

### Coding and analysis

Transcripts were coded using NVivo 11 (QSR International). Following the approach outlined by Guest et al. [22], the analysis team (J.T., S.P., and A.W.) first created a codebook of structural codes that corresponded to the abovementioned five main areas of inquiry. The team then developed emergent theme-based codes under these general domains including, for example, perceived positive and negative program features, cultural and gender issues, and problems with HVL use and how these problems were addressed [22]. Initially, two analysts (J.T. and S.P.) independently coded eight transcripts, then assessed intercoder agreement. Analysts reviewed inconsistencies, revisited the codebook to ensure appropriate code usage, and re-coded transcripts when emergent themes were identified. Remaining transcripts were divided between the two analysts for coding. A third analyst (A.W.) reviewed the analysis and provided quality assurance. We summarized themes from the general domains and present the most salient themes within each domain. In the results, we use “most” or “majority” to indicate more than 75% respondents said something; “many” indicates 50–75%; “some” or “several” represents 25–49%; and “a few” represents less than 25%.

## Results

We conducted six IDIs with CHSs and 47 IDIs with CHWs (Table 2), representing 93% of female and 63% of male CHWs in the pilot. Male CHWs were more challenging to recruit for IDIs due to other livelihood responsibilities. Of the 47 CHWs, five female CHWs (four in Herat and one in Kandahar) reported not having possession of a tablet following training and were excluded from analysis based on lack of implementation experience. Results are presented on HVL operational feasibility and acceptability of the HVL among CHWs.

**Table 2:** IDI participants by province

Participant Group	Balkh	Herat	Kandahar	Total
CHSs^a^				
Trained	2	2	2	6
Interviewed	2	2	2	6
CHW (male)				
Trained	10	10^b^	10	30
Interviewed	5	4	10	19
CHW (female)				
Trained	10	10	10	30
Interviewed	9	10	9	28

^a^All CHSs were male

^b^Male CHWs in Herat were not formally trained at the start of the pilot but received informal training during monitoring visits upon request

### Part 1: HVL operational feasibility

CHWs considered the pilot a success; however, they identified challenges and suggested solutions. CHWs provided insight into HVL operational feasibility based on the following: technology (i.e., tablet power supply and operations), capacity building and support (i.e., tablet use training and post-training support), program design (i.e., video format, topics, and languages), and scope (i.e., HVL reach).

#### Tablet power supply

The most commonly reported operational problem was maintaining tablet battery charge due to lack of electricity or readily available solar power. Many participants in Herat and Kandahar reported this issue but none in Balkh. CHWs described various solutions, including charging tablets at a neighbor’s home or a shop. When batteries depleted during meetings, CHWs reported counseling without tablets or ending meetings prematurely.

The tablets can keep little charge. Sometimes our meetings last for three hours, but the tablets cannot run that long, so we cannot show the entire program to people. (Male CHW, Herat)

#### Tablet operations

Most CHWs described the tablets as user-friendly, requiring little time to learn to operate. A few CHWs faced minor technical issues related to turning on the tablet, pausing videos, and playing videos in succession or in the correct language from a menu with no drop-down or branched components. Some female CHWs reported having little prior technology exposure and relied on family members or male CHWs for problem-solving.

#### Tablet use training

Overall, participants reported that training was useful and effective, made “our work easy,” and helped CHWs produce “positive results,” including increased client awareness of RMNCH issues. Several CHWs said training taught them how to operate and charge tablets. Technical issues were reportedly quickly resolved with guidance from a CHS, family member, or, less often, a peer CHW. A few CHWs and CHSs recommended the program provide repeat or extended training to ensure tablet use proficiency and implementation fidelity.

#### Post-training support

A few CHWs needed post-training support to improve skills, which they received either from CHSs, the program support phone number, or family members.

I had no information on how to use the tablet, but doctors trained me, and I somewhat learned how to use it. Since I had no experience using touch mobiles, I asked my family members to help me. Now I have no problem. (Female CHW, Herat)

A few male CHWs from all provinces said they knew to call their supervisor if they experienced trouble using tablets and seemed satisfied with this option. Other CHWs reported learning everything needed in the training but kept the support phone number in case problems arose.

#### Video format and topics

Low education and literacy levels, especially among female CHWs, were generally not barriers to HVL implementation given its audio-visual nature. For many CHWs, the HVL was easily understood and engaging because the visual videos were interesting, practical, and tangible. They felt the content, prepared as stories or songs, was easy to absorb and remember, especially for low literacy clients.

There is a proverb which states that hearing is not as good as seeing, so as they were hearing the audio and watching the video, that was very easy to understand for them. It was easy for me, too. (Male CHW, Balkh)

Many CHWs said all topics were easy to discuss and that post-video counseling sessions reinforced video content. When a sensitive topic did arise (e.g., family planning), most CHWs did not have trouble answering questions or explaining misconceptions.

#### Languages for video content

Most CHWs indicated having videos available in Dari and Pashto seemed adequate for reaching clients in different regions. A few participants thought having videos in more localized languages would be helpful, especially when discussing video content. This was illustrated by a female CHW from Balkh who said that if clients did not understand a video, they would ask her to repeat the lesson *“*from our own language.” CHWs reported that counseling in the local dialect was critical for ensuring client comprehension.

#### HVL program reach

Several CHWs reported reaching most women and community members with the HVL. A few male CHWs reached primarily men when accompanying their female counterparts to home visits and showing videos to male family members or mixed sex audiences. However, some CHWs and CHSs commented the HVL did not reach everyone in their catchment area. Reasons cited were as follows: geographically large or populous communities, insufficient numbers of CHWs or CHWs not having enough time, and hard-to-reach places. Participants recommended distributing more tablets and having more CHWs per community.

### Part 2: HVL acceptability among CHWs

CHWs generally reported HVL sessions were positive experiences. Many of the elements of acceptability related to how the HVL affected their duties. For example, CHWs discussed acceptability in terms of increased time efficiencies, reduced CHW information burden, and enhanced professional credibility and demand for services. However, some challenges were noted surrounding tablet possession, inability to meet heightened visit demand following HVL introduction, and issues related to movement restrictions.

#### Increased time efficiency with HVL

CHWs found the HVL made communication of technical RMNCH topics clear and concise. Some CHWs thought videos allowed clients to relate to issues more deeply than when receiving the same information verbally. Most CHWs felt that clients viewing videos typically understood lessons more quickly and without as much repetition, leading to time efficiencies for CHWs.

Women found videos interesting and said that [before the HVL] it was hard for them to keep the topics in mind, but watching videos helped them easily memorize the topics since they quietly sit and watch the videos. The videos made our job easy, too, since women learn more. (Female CHW, Herat)

#### Reduced CHW information burden

Many CHWs reported HVL use reduced the need to memorize large amounts of RMNCH information. Some CHWs also mentioned that videos prompted their recollection of critical information or they used the video’s story for reference during counseling. Several CHWs also commented that videos made their jobs and clients’ experiences more enjoyable. CHSs unanimously agreed the HVL helped CHWs more effectively convey health messages, making them clear and comprehensible.

#### Enhanced professional credibility and demand for CHW services

CHWs credited the HVL and associated counseling improvements with augmenting their credibility in their communities. Many CHWs reported the HVL improved clients’ trust of CHWs and affirmed their role. In tandem with enhanced professionalism, the HVL increased demand for CHW services, specifically for HVL sessions.

Women requested videos to be shown for their female guests or to other members of their family, such as daughters and in-laws, because they think videos are effective and easy to understand. (Female CHW, Herat)

### Challenges conducting duties exacerbated by HVL

Several positive effects attributed to the HVL also resulted in duty-related challenges for CHWs, particularly tablet possession and use, meeting client demand for sessions due to mobility restrictions, and cultural expectations around hosted gatherings. CHWs also had to resolve negative community perceptions about the HVL or tablet technology.

#### Tablet access and possession by gender

Although tablets were assigned to female CHW custody and meant to be shared within CHW pairs, many male CHWs in Kandahar took possession of the tablets. According to a few CHSs, this was because women were “weak-minded” and unable to keep tablets safe. Aside from Kandahar, CHWs and CHSs did not encounter tablet-sharing disputes. Sharing strategies included having designated days or transferring content to a male CHW’s personal device.

Although female CHWs kept the tablets, many acknowledged the benefit of sharing with men, including the HVL reaching male audiences. Several participants recommended providing more tablets to expand program reach and resolve gender-related access issues, especially since audiences were nearly always single-gender and men tended to view the HVL in other locations.

[Men] are going to the mosque and use it. Or, in session, my son is showing the videos to them. (Female CHW, Balkh)

#### Additional limitations on HVL coverage and session attendance

In addition to community size and competing CHW time demands, reach was hindered by conservative social norms restricting female mobility and thus session attendance. According to most CHWs who discussed mobility, women typically needed permission from their husband or another family member before visiting the health post. As such, many CHWs noted a marked increase in home visit requests by the husbands or clients themselves and reported difficulty in fulfilling these requests due to time limitations or restrictions to their own movement posed by the same norms. By contrast, many female CHWs mentioned that community leader and male partner support were critical for maximizing audiences and time efficiencies.

After men started to talk about this program in the community mosque, we see a lot of changes and all families let their women visit the health post. (Female CHW, Herat)

#### Unanticipated costs imposed by HVL demand

Despite perceived increased demand, several CHWs from all sites suggested providing financial incentives to encourage women to come to health posts rather than have CHWs incur costs associated with conducting multiple home visits to reach the same number of women. A few CHWs also mentioned incentives would help very poor women attend. Regarding increased session demand, CHWs mentioned unanticipated costs because clients generally expected refreshments at the gatherings.

#### Dispelling negative community perceptions and concerns

CHWs mentioned some initial community perceptions that hampered HVL use, including that CHWs were being paid to implement the HVL, that men should not be involved in women’s issues, or, in Kandahar particularly, that videos were inappropriate for their communities. Countering these perceptions required additional time and effort during the initial implementation period. According to a few CHWs, female clients in Kandahar and Herat expressed worry about being surreptitiously filmed or photographed during HVL sessions despite CHWs being told not to take pictures or videos of client sessions.

## Discussion

CHWs perceived the HVL intervention to be feasible and acceptable for their work in educating households on key RMNCH topics and services. The pilot demonstrated promise for improving CHW daily workload in diverse areas of Afghanistan through greater job efficiency and satisfaction. Notably, the HVL provided a portable complement to CHWs’ knowledge and skillsets. This is important in a predominantly rural context where timely information on complex issues can be limited. Moreover, this communication channel was acceptable for populations with little prior video exposure. The HVL engaged and informed low literacy clients through stories about complex RMNCH topics. Videos also helped focus counseling sessions, which reduced CHW time obligations. These efficiencies could ultimately lead to more clients attending a single session or more sessions within a given time frame. Moreover, the reduced technical information burden allowed CHWs to focus on consistent messaging, potentially resulting in higher counseling quality available to larger numbers of clients.

These findings are similar to studies from other contexts, including South Africa and India, where tablet- and mobile phone-based HVL pilots reported that mobile technologies were useful tools that improved CHW engagement with clients [17, 18]. A study from Colombia also found that CHWs with little formal education and training could leverage mobile devices to improve engagement and their overall quality of community-based care [23]. These improvements may make CHWs more productive; indeed, in India, mobile videos contributed to more efficient use of CHWs’ time [19]. In Malawi, mHealth tools allowed CHWs to double the number of clients they served [24]. In addition, our findings suggest that the HVL pilot alleviated some pressures caused by time poverty, a challenge also reported by CHWs in Rwanda [25]. As CHW turnover yields new recruits with incomplete training in Afghanistan, the HVL may be an important mechanism to support a range of training and knowledge acumen within new and active CHWs. Even CHWs not proficient with mobile technologies prior to the pilot found the tablets easy to use with refresher training or informal support.

Regarding feasibility, some provider-level operational challenges should be addressed prior to expanding HVL implementation. The primary issue was an unreliable electricity source, especially in Herat and Kandahar. This problem was reported less frequently in Balkh, potentially due to its proximity to Central Asian Republics from which northern Afghanistan receives power. Tablets have an 8-h battery life, but large applications, including video players, demand considerable power. CHWs were counseled to limit tablet use to HVL sessions to save battery. Solar chargers were not supplied, but scale-up efforts should consider distributing these within the standard HVL package. Alternatively, they could be provided as an incentive to motivate CHW performance.

Limited tablet supply was also a concern, as many CHWs requested tablets for both men and women to ensure adequate coverage. In many communities, male and female CHWs serving at a health post are relatives, frequently married couples, and tablet-sharing seemed feasible at program design. However, wider tablet distribution could attenuate gender-based issues and expand HVL coverage. It may not be financially feasible to supply all CHWs with a device; CHSs should moderate tablet-sharing, ensuring the CHWs they manage adhere to a tablet custody schedule. Making verbal agreements to such a schedule with the CHS and *shura* might encourage stricter adherence. Separately, CHWs with smart phone access could transfer video content to their personal device to facilitate counseling sessions in lieu of a tablet.

We recommend that scale-up efforts include refresher trainings to ensure CHWs feel adequately supported using the HVL, similar to calls for expanded CHW training and support in other settings [26, 27]. Implementing mobile technologies in low-resource settings also raises concerns about appropriate tablet management, and scale-up training should emphasize practices that extend battery life. Trainings should also highlight strategies for using tablets to optimize counseling sessions, including pausing videos to invite dialogue or referring to specific segments to reinforce messaging. Providing regular training may further support HVL use and ensure adequate return on investment for the HVL, given the relatively high initial cost.

The longer-term challenges to scaling up the HVL relate to socio-cultural barriers and limitations of the CHW model that may be more difficult to address. Gender issues were a prominent emergent theme that requires engagement with traditional leadership and program design considerations. For female CHWs and their clients, addressing gender norms with community leaders prior to HVL introduction may be essential in a context where many women cannot move freely outside the home or make their own health decisions [4]. Many women required permission to attend HVL sessions at health posts, increasing CHW workload through travel required for home visits. Costs associated with transportation and a male escort (*maharam*) may make these visits impossible for volunteer workers. In some cases, promoting the HVL to men improved women’s opportunities to attend health post-based sessions. This engagement approach should be conducted in tandem with community leader advocacy to optimize session attendance at health posts.

Tablet ownership and male client exposure were primary concerns resulting in male CHWs in Herat being trained after pilot inception. Engaging male CHWs, while useful for male clients, had some negative implications for female CHWs, especially in Kandahar where tablets were withheld. Based on similar feedback, we modified the intervention for scale-up to include tablets for male CHWs. More equal tablet distribution may build greater respect for all CHWs, potentially helping to address credibility issues experienced by some CHWs in Afghanistan [4]. In South Africa, CHWs reported increased self-confidence through tablet use [28], and nurse midwives in India felt using mobile phone videos improved their perceived authority and patient trust [18]. We found similar improvements in CHW perceptions of community trust and respect. CHWs felt their status was elevated through the HVL and that clients were more willing to listen.

### Limitations

Social desirability bias may be present as CHWs potentially exaggerated their satisfaction with or their performance implementing the HVL. Although IDIs were conducted in a private room, interviews occurred at a refresher training where supervisors and facilitators were present. This may have limited CHWs’ comfort in relaying any negative experiences or inspired more positive reporting. However, given the many challenges reported, we do not consider this to be a significant threat to the validity of our findings. Another challenge was that interviewers did not consistently ask follow-up questions within all sections of the interview, particularly around gender issues. Given that gender issues were an emergent theme, we have limited ability to fully describe difficulties with implementation caused by gender-related concerns. Although our mixed methods approach aimed to obtain larger samples of key feasibility and acceptability measures among the target audiences and contextualize those findings with qualitative data from some key influencers, the IDI population may have benefited from broader representation. Specifically, mothers-in-law may act as gatekeepers to HVL access and their perceptions and influence were not gauged, though this may have bearing on engagement with CHWs and HVL exposure. Finally, we did not collect socio-demographic data from CHWs, which may have been useful for further contextualizing the results.

### Conclusions

We selected culturally and geographically diverse provinces as pilot sites to ensure that lessons learned would be broadly applicable to other areas of the country. We found that the HVL pilot was highly acceptable and feasible for integration into existing duties among CHWs, indicating it may be effective for improving RMNCH knowledge and care-seeking behaviors across Afghanistan. Notwithstanding, we identified operational challenges that should be addressed to ensure successful intervention scale-up to remote areas or those with a deficit of providers. Issues with technology may be easily addressed through ongoing training, whereas hardware issues could be improved using solar chargers or adopting practices that extend battery life.

Longer-term operational challenges, including tablet distribution and community reach, require greater consideration. With a national workforce of more than 20,000 CHWs [4], inequitable table distribution may exclude certain communities. Implementers must therefore prioritize areas of greatest need at the provincial level but also strategize with local leaders to build support for HVL group-based counseling to the extent possible. This pilot also highlighted gender-related challenges with tablet distribution, though deeper analysis is necessary to understand how these will inform HVL roll-out. As a first step, male CHWs should have equal access to tablets or HVL content, as male engagement also proved beneficial for female clients. As implementers work to address operational issues, we are encouraged that the scale-up of this technology will expand access to much-needed RMNCH knowledge and care for hard-to-reach populations across Afghanistan.

## Data Availability

The data generated and analyzed for this study are available from the corresponding author on reasonable request.

## Abbreviations

CHS: Community health supervisor
CHW: Community health worker
HEMAYAT: Helping Mothers and Children Thrive in Afghanistan
HVL: Health video library
IDI: In-depth interview
MOPH: Ministry of Public Health
RMNCH: Reproductive, maternal, neonatal, and child health
